# Hospice at Home services in England: a national survey

**DOI:** 10.1136/bmjspcare-2019-001818

**Published:** 2019-11-13

**Authors:** Melanie Rees-Roberts, Peter Williams, Ferhana Hashem, Charlotte Brigden, Kay Greene, Heather Gage, Mary Goodwin, Graham Silsbury, Bee Wee, Stephen Barclay, Patricia M Wilson, Claire Butler

**Affiliations:** 1 Centre for Health Services Studies, University of Kent, Canterbury, UK; 2 Department of Mathematics, University of Surrey, Guildford, UK; 3 Hospice at Home, Pilgrims Hospices in East Kent, Canterbury, UK; 4 Research Lead, National Association for Hospice at Home, Fareham, UK; 5 Hospice at Home, Mary Ann Evans Hospice, Nuneaton, Warwickshire, UK; 6 Surrey Health Economics Centre, University of Surrey, Guildford, UK; 7 Palliative Medicine, Oxford University Hospitals NHS Trust, Oxford, UK; 8 Department of Public Health and Primary Care, University of Cambridge, Cambridge, UK

**Keywords:** End of life, hospice at home, palliative care, services, hospice

## Abstract

**Objective:**

Hospice at Home (HAH) services aim to enable patients to be cared for and die at home, if that is their choice and achieve a ‘good death’. A national survey, in 2017, aimed to describe and compare the features of HAH services and understand key enablers to service provision.

**Methods:**

Service managers of adult HAH services in the ‘Hospice UK’ and National Association for Hospice at Home directories within England were invited to participate. Information on service configuration, referral, staffing, finance, care provision and enablers to service provision were collected by telephone interview.

**Results:**

Of 128 services invited, 70 (54.7%) provided data. Great diversity was found. Most services operated in mixed urban/rural (74.3%) and mixed deprivation (77.1%) areas and provided hands-on care (97.1%), symptom assessment and management (91.4%), psychosocial support (94.3%) and respite care (74.3%). Rapid response (within 4 hours) was available in 65.7%; hands-on care 24 hours a day in 52.2%. Charity donations were the main source of funding for 71.2%. Key enablers for service provision included working with local services (eg, district nursing, general practitioner services), integrated health records, funding and anticipatory care planning. Access to timely medication and equipment was critical.

**Conclusion:**

There is considerable variation in HAH services in England. Due to this variation it was not possible to categorise services into delivery types. Services work to supplement local care using a flexible approach benefitting from integration and funding. Further work defining service features related to patient and/or carer outcomes would support future service development.

## Background

Demographic studies predict a future of increasing numbers of older people and increasing numbers of deaths.^1^ The number of people wishing to die at home is also increasing,^1–4^ however, evidence about whether this preference changes as illness progresses is mixed.^4 5^ The provision of home-based support services at the end of life increases the number of people able to die at home.^6^ These services are highly rated by referring healthcare professionals who cite the support, time and experience provided enabling patients to die at home.^7^ Identifying how care can be delivered and maintained at home was identified as a top 10 priority by the James Lind Alliance in 2015 and providing patients with choice about where they receive their care at the end of life is central to UK policy.^8 9^

Hospice at Home (HAH) services aim to offer the quality and ethos of hospice care, at home, to support dying patients to have a ‘good death’ in their place of preference. The National Association for Hospice at Home (NAHH) has recommended six core, national standards for HAH services, developed with stakeholders to support the delivery of HAH services.^10^ Having been established across England since the development of the ‘modern hospice movement’ in the late 1960s, HAH services tend to share a number of characteristics. These include: the aim to enable patients to be cared for and die in their place of choice if that is their own home; employing ‘specialist’ staff with high levels of palliative care experience and having the ability to provide more staff time with the patient than pre-existing/other services. Many are run or contributed to by independent charities which have designed a HAH service to suit the needs and setting of the local population.

This natural evolution of HAH services designed to meet local need presents an opportunity to understand at a national scale whether particular elements of service provision impact on patient outcomes and care costs in order to inform national policy. National healthcare providers and policymakers require evidence on how to provide quality, outcome and experience-based, cost-effective, end-of-life care across the country. With an ageing population and a renewed focus on primary and community services in the National Health Service (NHS) Long-Term Plan,^11^ evidence on how to provide integrated end-of-life care in the home tailored to local populations is critical.

The NAHH, working with Hospice UK, conducted a survey in 2012 across 76 HAH services in England.^12^ This indicated there to be at least two types of service model,^12^ those that delivered high numbers of episodes of care versus services that offered significantly fewer, with notable differences between the two (eg, reasons for referral, duration of episodes, who is involved in delivering care and knowledge regarding preferences and place of death). This provided useful data to start to describe the landscape of HAH and concluded that more than one model of service exists, and they are not homogenous in their outcomes.^12^ Further understanding of the range and variation in existing HAH services is needed before assessing how best to deliver effective services at scale and in a cost-effective manner to achieve the outcomes desired. This paper reports a survey that aimed to describe and compare the features of HAH services in England and understand key enablers to service provision.^13^

## Methods

This study followed a mixed methods, convergent parallel design combining quantitative survey data to identify HAH service features with quantitative and qualitative data on enablers and barriers of service provision.

Service managers from HAH services in England were invited to take part in a telephone survey. Adult HAH services in England were identified from the ‘Hospice UK’ Service Directory and cross-referenced with the NAHH databases (both received 28 October 2016, n=128). Service managers were contacted by post with an invitation letter providing details of the research, an opt-out slip and a copy of the telephone survey (online supplementary material 1). Service managers were then contacted after 2 weeks to arrange a telephone appointment to collect data.

10.1136/bmjspcare-2019-001818.supp1 Supplementary data

All data were collected during a 5-month period (February 2017 until July 2017) by an experienced palliative care nurse with knowledge of the setting to obtain contextually accurate data. Services could return the opt-out form if they did not wish to take part and if services had not opted out, no more than three attempts were made to arrange a telephone appointment for data collection. Reasons for not taking part in the study were not requested.

The survey questionnaire consisted of closed questions covering topics including service setting (location, geographical area type, deprivation, population served and other supporting services locally), referral numbers and criteria, services provided (rapid response services, types of care provided), hours of service, patient use of services (duration and intensity of service use by patients), staffing (roles, number of staff and full-time equivalent data) and to what extent defined factors supported the provision of the service and funding (eg, support and relationships with commissioners and other services, workload and funding, staffing, access to medication or equipment to provide services and geographical challenges). During the survey questions involving factors supporting the provision of services, field notes were collected in parallel to survey data and used as qualitative data. Notes consisted of detailed summaries of significant enablers or barriers discussed with interviewees.

The interpretation of the survey findings involved iterative discussion work with the wider collaborative team including lay coapplicants with experience of HAH care. Survey responses were analysed using descriptive statistics and SPSS software (V.15) and all findings presented in tables (online supplementary material 2). Categorical variables (eg, setting urban/rural) were cross-tabulated with each other in order to identify underlying associations. Continuous variables (eg, population served) were compared between different categories of each categorical variable, as well as being plotted against each other, in order to identify underlying associations. These results were used to identify any natural groupings of service features that could be defined as service models or types.

10.1136/bmjspcare-2019-001818.supp2 Supplementary data

Qualitative field notes were typed up and analysed inductively to identify important context and environmental elements of service provision.^14^ These service-enabling or barrier elements were discussed and findings finalised by the research team at the University of Kent before being refined further by the wider collaborative research team.

## Results

One-hundred and twenty-eight HAH services in England identified from the NAHH and Hospice UK directories of services were approached to take part in the survey, 113 (88%) were charity-led and 15 (12%) NHS-led services. Over 5 months, survey data were collected from 70 HAH services (54.7% response rate). Twenty-two services opted out of the survey and a further 36 services could not be contacted after three attempts. The postcode for each hospice was collected to pinpoint their geographical location. Postcodes were mapped and responders and non-responders compared for their geographical location to ensure wide geographical spread of both groups.

### Service size and setting

Responding HAH services represented a wide range of size (based on referrals per annum). The mean number of referrals per annum was 452, with a minimum service size of 62 referrals per year and maximum of 2222 referrals (SD 393.7, IQR 405). Geographical area ranges covered by some services were large (across counties), serving total populations ranging from 5000 to 1.2 million (median 249 000; IQR 270 250). On average, 2.5 referrals were received per 1000 of the total population annually (SD 2.8, IQR 2.3).

Table 1 details the setting in which HAH services operated. Most services operated in mixed urban and rural settings (n=52, 74.3%) and across areas with mixed deprivation levels (n=54, 77.1%). Just 10.0% of services (n=7) provided HAH support in solely urban areas and 15.7% (n=11) among only rural communities. Few HAH services were running in predominantly deprived areas (n=5, 7.1%), while 11 services (15.7%) operated in predominantly affluent areas only. When asked whether the geography of the area made it difficult to provide services, many responders (n=61, 87.1%) thought this factor made service provision somewhat or substantially challenging.

**Table 1:** HAH service setting (n=70 HAH services)

HAH service setting	Hhospices n (%)
Geographical area	
Rural	11 (15.7)
Urban	7 (10.0)
Mixed	52 (74.3)
Level of deprivation	
Predominantly deprived	5 (7.1)
Mixed deprivation	54 (77.1)
Predominantly affluent	11 (15.7)
Local 24 hours’ district nursing	54 (78.3)
Operating alongside other HAH services	18 (25.7)
Access to Marie Curie services	49 (70.0)

HAH, Hospice at Home.

Most services operated alongside 24 hours’ district nursing NHS services (n=54, 78.3%) and alongside community specialist palliative care services (n=61, 87.1%). Just over one-quarter were operating alongside other HAH services in the same area (n=18, 25.7%) and 70% (n=49) with access to local Marie Curie services.

### Referral criteria

Services had highly variable referral criteria with respect to the life expectancy of patients accepted for HAH care (table 2). Twelve services (17.1%) provided care solely for actively dying patients, defined as having hours/days or up to 2 weeks to live. More services accepted referrals from patients within medium-term prognoses of within the last 6 months of life (n=18, 25.8%), however, most services had less strict cut-offs for referral, accepting patients with over 6 months’ prognosis with no upper boundary (n=40, 57.1%). When asked if the referrals made to the service were manageable and appropriate, most service managers expressed that the referrals received were somewhat or substantially manageable and appropriate for their service (n=68, 97.1%).

**Table 2:** HAH service life expectancy referral criteria (n=70 HAH services)

Referral life expectancy	Hospices n (%)
Actively dying only—within hours/days	1 (1.4)
Up to last 2 weeks of life	11 (15.7)
Up to last month of life	9 (12.9)
Up to last 3 months of life	7 (10)
Up to last 6 months of life	2 (2.9)
Up to last year of life	12 (17.1)
>12 months to live	28 (40)

HAH, Hospice at Home.

### Services provided

Table 3 details the types of care provided by HAH services. Most services provided personal hands-on care such as washing or direct personal care for the patient (n=68, 97.1%), symptom assessment and management (n=64, 91.4%) and psychosocial support for patients and/or family carers (n=66, 94.3%). Two-thirds of services also provided respite care to support carers (n=52, 74.3%). Approximately half of HAH services were able to provide care 24 hours a day and 7 days a week (24/7). Fewer services (n=15, 21.4%) provided practical support (household tasks, eg, shopping) directly for family members or carers.

**Table 3:** Types of HAH care (n=70 HAH services)

Type of care	Services n (%)	Hours care provided				
		24/7 n (%)	08:00–20:00 Monday to Sunday n (%)	09:00–17:00 Monday to Sunday n (%)	09:00–17:00 Monday to Friday n (%)	Other n (%)
Hands-on personal care	68 (97.1)	35 (52.2)	9 (13.4)	6 (9.0)	2 (3.0)	15 (22.4)
Symptom assessment and management	64 (91.4)	39 (60.9)	9 (14.1)	1 (1.6)	3 (4.7)	12 (18.8)
Psychosocial support	66 (94.3)	40 (60.6)	6 (9.1)	4 (6.1)	7 (10.6)	9 (13.6)
Practical support at home	15 (21.4)	2 (2.9)	5 (7.1)	1 (1.4)	4 (5.7)	3 (4.3)

	Services n (%)	24/7 n (%)	Day n (%)	Night n (%)	Other n (%)	
Respite care	52 (74.3)	33 (52.2)	9 (13.4)	6 (9.0)	2 (3.0)	

HAH, Hospice at Home.

Many HAH services were able to provide rapid response times (including at weekends) for patients requiring care, with 65.7% of services (n=44) able to respond to a patient within 4 hours of contact, 29.9% (n=20) able to respond within 24 hours and the remaining (n=3, 4.5%) responding the next working day.

Over half of services (n=36, 60.0%) cared for patients between 1 week and 2 months on average once referred. Fewer provided care for less than 1 week (n=9, 15.0%) and slightly more services provided care for over 2 months (n=15, 25.0%). Although asked as a question, intensity of care data were difficult for service managers to provide as this was not routinely collected. Data obtained indicate that half of services (n=32, 50.0%) provided intensive care to patients (more than 3 hours/day) daily. Many services also had local access to inpatient palliative care beds if required (n=66, 94.3%) in either a hospice, hospital or care home setting.

When asked about factors that made it difficult to provide HAH services, the inability to access necessary equipment and anticipatory medicines in a timely fashion proved difficult for more than half of HAH services (n=39, 55.7%). Furthermore, the delay in being able to administer anticipatory medicines by injection in a timely fashion also caused service delivery difficulties (n=43, 61.4% of HAH services reporting this as somewhat or substantially difficult to provide). Many service managers felt HAH received substantial non-monetary support from local commissioners, the hospices themselves, community nurses and general practitioners (98.1%, 98.6%, 100.0% and 100.0%, respectively).

### Staffing

Staffing data proved difficult to collect and analyse as many HAH services could not provide accurate data over the telephone at the time of survey. Therefore, type of staff employed by HAH services in whole-time equivalents was not possible to deduce. On average, HAH services employed 19 members of staff (SD 10.84, minimum 1 and maximum 51) with a large range of staffing roles and models across HAH services surveyed (table 4). More than half (n=37, 52.6%) of services had at least three or more different staff disciplines illustrating the multidisciplinary nature of HAH care services. Nearly all services employed registered nurses and/or healthcare assistants (HCA) to provide day-to-day care (n=66, 98.6%). Many services (n=45, 66.2%) did not employ additional staff solely dedicated for HAH services, instead supporting front-line care staff (HCAs and/or nurses) with clinicians and healthcare professionals working across hospice and/or NHS services where needed. When asked if it was difficult to recruit and retain staff for HAH services, there was no difficulty for half of responders (n=38, 55.9%), while the remaining responders found it somewhat or substantially difficult to recruit and retain staff (n=28, 41.2% somewhat difficult and n=2, 2.9% substantially difficult, respectively).

**Table 4:** Service staff dedicated solely to HAH (n=70 HAH services)

Dedicated staff discipline	HAH services n (%)	Mean number of staff per HAH service n (SD, min–max)
Healthcare assistant	59 (86.8)	9.13 (7.68, 0–40)
Registered nurse	58 (85.3)	5.55 (4.79, 0–22)
Medical consultant or other doctor	18 (26.5)	0.36 (0.69, 0–3)
Physiotherapist	17 (25)	0.28 (0.51, 0–2)
Occupational therapist	15 (22.1)	0.24 (0.46, 0–2)
Counsellor	22 (32.4)	0.54 (0.97, 0–4)
Social worker	9 (13.2)	0.15 (0.40, 0–2)
Chaplaincy	15 (22.1)	0.24 (0.46, 0–2)
Volunteers	26 (38.2)	8.79 (30.26, 0–220)
Administrators	47 (69.1)	1.18 (1.24, 0–5)
Management (all registered nurses)	61 (89.7)	1.10 (0.69, 0–4)
All staff (not including volunteers)	n/a	19 (10.84, 1–51)
Missing	2 (2.9)	n/a

HAH, Hospice at Home; n/a, not applicable.

### Funding

**Figure 1:**
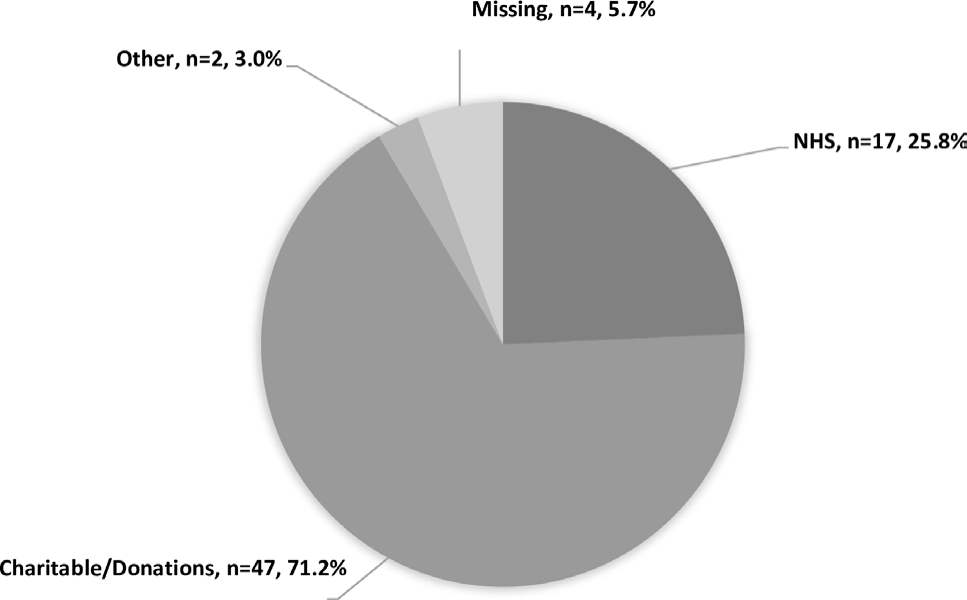
Main source of funding for Hospice at Home services (n=70 HAH services). This figure indicates the main sources of income supporting HAH services in England. Four services (labelled missing) did not give an answer to this question stating equal funding sources. HAH, Hospice at Home; NHS, National Health Service.

Service managers were asked about the sources of funding received to directly support the HAH service provided to patients. The majority of services were funded using charitable funds or donations as their main source of income (n=47, 71.2%, figure 1). One-quarter of services (n=17, 25.8%) received NHS funding as their main source of income with three services (4.5%) fully NHS or local authority funded. Four HAH services did not provide a main source of income, with three of these specifying the NHS as one of three or more sources of income. Many HAH services (n=44, 62.9%) received NHS funding as a secondary source, however, nine services (12.9%) received no NHS funding at all. When asked if having inadequate funding made it difficult to provide HAH services, 84.3% of responders indicated it made service provision somewhat or substantially difficult while 12.9% felt inadequate funding did not impact on service provision.

### Views on enablers for HAH services

The survey asked service managers about elements that supported provision or made it difficult to provide HAH services in their area. Notes recorded during survey interviews provided further insight into key contributing features for successful services. First, planning and integration of services locally was a major factor contributing to the provision of HAH services. Having a detailed business plan for commissioning and integration with other local end-of-life services enabled HAH service provision and funding. Furthermore, direct access to NHS Trust services or other suppliers of medication and equipment, as well as suitably trained and prepared people to undertake medication administration, was a key factor to patient care remaining within the home. The presence of an integrated patient record also allowed better integration and facilitated arrangement of anticipatory prescribing and advance care planning across providers.

Workforce, staff skills and wider support also emerged as key to supporting HAH services that allowed patients to die at home. Service managers expressed the need to have a service able to respond to changes in demand as patients could deteriorate at any time in the last hours-days-weeks of life and their resulting service needs fluctuate accordingly. Using a skilled workforce mix of permanent and flexible staff (under 0-hour contracts) enabled services to adapt to demand. Many service managers also reported that identifying patients requiring rapid response or intensive support using trained triage staff and being able to communicate the support available to patients and families was a key feature of success. HAH services also benefited from a well-trained and extensive network of third sector support, volunteers and a responsive family support system.

## Discussion

The results of this survey provide the first detailed description of the range of HAH service provision existing in England. The data show that HAH services work alongside local district nursing and other palliative services. They report varied levels of activity, staffing configurations and referral criteria. While almost all HAH services provide personal care, psychosocial support and symptom management, not all were able to provide this 24/7 or to offer respite care. Two-thirds of services reported charity donations as the main source of funds.

The goal of the survey was to understand the current national landscape and identify ‘models’ of provision for further investigation of outcomes and costs. Unlike a previous survey of HAH services in 2012, which indicated at least two types of service model,^12^ the heterogeneity within this sample meant services could not be clustered. This suggests that services may have changed provision, possibly in response to the rapidly changing commissioning landscape, adapting to provide more diverse and flexible services. Since this previous survey, services may also have undergone mergers, ceased to function or new services arisen to cover local unmet need. Broad groupings were defined based on annual referrals (less than or greater than/equal to 365), and whether or not the service offered care 24/7. Services from each were invited to be case study sites for in-depth evaluation of outcomes and costs.^13^ This follow-on study will produce guidelines and recommendations for commissioners and service providers by exploring what works for whom and under what circumstances in achieving a ‘good death’ at home if this is the patient’s preference.

Alongside the detailed picture on specific service features, evidence on key enablers gathered during survey interviews identified a number of critical elements that may contribute to successful services which will form important lines of investigation in the follow-on study.^13^ These views concurred with the six core NAHH standards, developed to support the delivery of HAH services nationally.^10^ Using a skilled workforce mix of permanent and flexible staff (under 0-hour contracts) enabled services to adapt to fluctuating patient demand. This feature aligns with the first and fifth NAHH standards for HAH services: workforce management, education and development strategy that ensures the competence and confidence in practice and a service that meets the assessed need for patients, carers and their families.^10^

Integration of local end-of-life services, the second NAHH national standard, was also a large contributing factor identified in this study as enabling HAH service provision. Direct access to an integrated patient record as well as skilled staff, medicines and equipment from local NHS Trusts or other services facilitated the provision of HAH services considerably.^15^ Despite many HAH services (77%) operating alongside 24 hours’ district nursing services in this study, out-of-hours, anticipatory prescribing and equipment provision have been highlighted as key areas requiring improvement.^15 16^ Adequate funding, collaboration and integration of services could enable wider access to specialist palliative care services at home for the benefit of patients and their carers.

HAH national standards (3, 4 and 5) advocate clearly defined and communicated referral criteria and pathways alongside service information enabling patients, carers and families to make informed choices and receive care that meets need.^10^ Identifying patient needs, including rapid response or intensive support, was identified by many service managers as a key feature of the success of their service. Existing evidence suggests that provision of a rapid response service increases the chances of patients dying at home if that is their preference as does increased public awareness of the HAH services.^17–19^

The final NAHH standard promotes systems and processes to ensure pre/post-bereavement support for patients (where appropriate), carers and families. Views on enablers from respondents to the survey identified that services benefited from a well-trained and extensive network of third sector support, volunteers and family support. Studies evaluating the carer’s perspective of HAH services have shown that these services are well received and can have a positive impact on carer well-being^2 16 19 20^; however, additional bereavement support was identified as a key area of need.^17^

## Limitations

This survey only included HAH services registered on the NAHH and Hospice UK databases. Therefore, there may be community and private services operating HAH services that were not included in this study. A 55% participation rate was achieved. Of the 128 services, 22 (17.2%) declined the invitation to be interviewed, and 36 (28.1%) could not be contacted. The reasons for non-participation are not known, but are likely due to the time involved in taking part in the interview or it is possible that some services listed in the directory had ceased to function or merged with others. Data collection for some key information (eg, staffing in whole-time equivalents) proved difficult resulting in varying amounts of missing data which has limited the analyses.

## Conclusion

This national survey highlights the considerable variation in features of HAH services in England. These services, funded largely by volunteer effort and charity donations, work alongside formal health and care provision in local areas of England to provide greater choice of palliative care at home. With such variation in services, understanding what features lead to improved patient and carer outcomes would support service provision in the future. Ongoing in-depth evaluation of 12 different HAH services that participated in this survey will explore what service features and processes work best for patients and carers in different contexts, and the resource implications, in order to provide recommendations for commissioners and providers in the future.^13^

## Data Availability

All quantitative data relevant to the study are included in the article or uploaded as supplementary information. Qualitative fields note are available subject to request.
