# Supplementary material for: Hospice at Home services in England: a national survey
Source: BMJ Support Palliat Care. 2019 Nov 13;11(4):454–60. doi: 10.1136/bmjspcare-2019-001818 (PMC8606452; doi:10.1136/bmjspcare-2019-001818)
Supplement: Supplementary data [file bmjspcare-2019-001818supp001.pdf]

OPEL H@H study – PHASE 1 SURVEY

Date of completion:

Hospice at home service (title):

Verbal consent confirmed and survey completed by:

……………………………………………………………………….(researcher)

In discussion with

……………………………………………………………..(name)…………………………………….(job title) from the service.

Geography/demographics

Location of service	(town (s), county):
Type of geographical area	Urban/ rural/ mixed
Levels of deprivation in the area?	Predominantly deprived/mixed/predominantly affluent
Total population served (if known)	number

Other services operating in the area

Are there other hospice at home services operating in the same area?	Yes/no
District nursing services in the area	24h/<24h
Community specialist palliative care service(s)	Yes/no
Marie Curie service	Yes/no

Inpatient palliative care beds

Do patients living in the area covered by your service have access to inpatient palliative care beds? Yes/No .
If Yes:-

	number
In hospice	
In hospital (designated palliative care beds/unit)	
In community hospitals	
In care/nursing homes	

The OPEL H@H project is funded by the NIHR
Health Services and Delivery Research Programme

Understanding your HAH service

What are the referral criteria for your HAH service *based on prognosis*?

Service criteria –	***Tick all which apply***
Actively dying – within hours/days	
last 2 weeks of life	
last month of life	
last 3 months of life	
last 6 months of life	
last year of life	
Known to have life limiting illness, anticipated life expectancy greater than 12 months	
Other<br>*specify*	

Does your HAH service provide a "rapid response"?

If an urgent referral is received at say 11pm (2300) on a Friday evening, what is the response time for a visit from the service?	* within 4 hours<br>* within 24 hours<br>* next working day Mon-Fri

What type of services does your HAH service provide?

Service categories	***Tick all which apply***	***When is this element of the service available?***
Personal hands on care		* 24/7<br>* 8am-8pm, 7 days a week<br>* 9am-5pm, 7 days a week<br>* 9am-5pm, Mon-Fri
Symptom assessment and management – Physical		* 24/7<br>* 8am-8pm, 7 days a week<br>* 9am-5pm, 7 days a week<br>* 9am-5pm, Mon-Fri

The OPEL H@H project is funded by the NIHR
Health Services and Delivery Research Programme

NHS
National Institute for
Health Research

Psychosocial support for Patient and/or family carers		* 24/7<br>* 8am-8pm, 7 days a week<br>* 9am-5pm, 7 days a week<br>* 9am-5pm, Mon-Fri
Respite care visits -		*Day*<br>*Night*<br>*Both*
Practical support for family carers<br>*(different to personal care e.g. housework)*		* 24/7<br>* 8am-8pm, 7 days a week<br>* 9am-5pm, 7 days a week<br>* 9am-5pm, Mon-Fri
Other – please specify		

HAH service clinical activity data

Over **the past calendar year (or financial year)** can you estimate the following information?

The number of referrals to the service	number
The duration of service use for most of your patients-	* <1 week<br>* 1 week – 2 months<br>* > 2 months
The intensity of service use for most of your patients	* < 3h care/week<br>* between 3h care/day – 3h care/week<br>* > 3h care/day<br>* a roughly even split of the above

HAH service Staffing

How many staff members in each of the following categories do you have **dedicated entirely to the HAH service?**

Category	Number of staff	Whole time equivalent (WTE) if category is NOT all full-time staff	Any comments
Healthcare assistants			
Registered nurses			

The OPEL H@H project is funded by the NIHR
Health Services and Delivery Research Programme

NHS National Institute for Health Research

Medical Consultants			
Other doctors			
Physiotherapist			
Occupational therapist			
Counselling staff			
Social work staff			
Chaplaincy staff			
Volunteers			
Administrative staff			
Management			
Other – please specify _______________________			

Enablers and barriers to providing the defined HAH service

To what extent do each of these factors SUPPORT you in running the service you aim to provide?

FACTOR EXTENT:	NOT AT ALL	SOMEWHAT	SUBSTANTIALLY
Support from local commissioners (clinical commissioning group)			
Support from Board of Trustees of charity			
Relationship with local hospice			
Relationship with local generic community nursing services			
Relationship with local GPs			
Relationship with other local service – please SPECIFY _________________________			
Manageable number of referrals			
Referrals are suitable/appropriate for the service			
adequate funding			

The OPEL H@H project is funded by the NIHR
Health Services and Delivery Research Programme

NHS National Institute for Health Research

ability to recruit and retain suitable staff			
Other services (e.g. social services, continuing healthcare funded services) provide care in a timely fashion			
Ability to provide services out of hours			
Ability to access necessary clinical equipment in a timely fashion			
Ability to access anticipatory ("just in case") medications by injection in a timely fashion			
Ability to provide administration of anticipatory ("just in case") medications by injection when needed			
Geography of your area (e.g. distances, parking, traffic, safety)			

To what extent do each of these factors MAKE IT DIFFICULT to run the service you aim to provide?

FACTOR EXTENT:	NOT AT ALL	SOMEWHAT	SUBSTANTIALLY
Lack of support from local commissioners (clinical commissioning group)			
Lack of support from Board of Trustees of charity			
Relationship with local hospice			
Relationship with local generic community nursing services			
Relationship with local GPs			
Relationship with other local service – please SPECIFY ________________________			
Too many referrals			
Unsuitable/inappropriate referrals			
Inadequate funding			
Inability to recruit and retain suitable staff			
Difficulty getting other services (e.g. social services, continuing healthcare funded services) to provide care in a timely fashion			

The OPEL H@H project is funded by the NIHR
Health Services and Delivery Research Programme

NHS
National Institute for Health Research

Difficulty providing services out of hours			
Difficulty accessing necessary clinical equipment in a timely fashion			
Difficulty accessing anticipatory ("just in case") medications by injection in a timely fashion			
Delays in administration of anticipatory ("just in case") medications by injection when needed			
Geography of your area (e.g. distances, parking, traffic, safety)			

Funding

How is the HAH service funded?

Main source of income	NHS OR charitable OR donations OR other – please SPECIFY________________________________
Other sources of income (tick all that apply)	* NHS<br>* charitable<br>* donations<br>* other – please SPECIFY__________________________

The OPEL H@H project is funded by the NIHR
Health Services and Delivery Research Programme

NHS National Institute for Health Research
