# Supplementary material for: Hospice at Home services in England: a national survey
Source: BMJ Support Palliat Care. 2019 Nov 13;11(4):454–60. doi: 10.1136/bmjspcare-2019-001818 (PMC8606452; doi:10.1136/bmjspcare-2019-001818)
Supplement: Supplementary data [file bmjspcare-2019-001818supp002.pdf]

OPEL SURVEY. Frequency Tables (N=70 respondents)

Date of completion of Survey (MONTH)

		Frequency	Percent	Valid Percent	Cumulative Percent
Valid	2	2	2.9	2.9	2.9
	3	25	35.7	35.7	38.6
	4	17	24.3	24.3	62.9
	5	19	27.1	27.1	90.0
	6	7	10.0	10.0	100.0
	Total	70	100.0	100.0	

Date of completion of Survey (YEAR)

		Frequency	Percent	Valid Percent	Cumulative Percent
Valid	2017	70	100.0	100.0	100.0

Type of Geographical area

		Frequency	Percent	Valid Percent	Cumulative Percent
Valid	Urban	7	10.0	10.0	10.0
	Rural	11	15.7	15.7	25.7
	Mixed	52	74.3	74.3	100.0
	Total	70	100.0	100.0	

Urban extent of locality

		Frequency	Percent	Valid Percent	Cumulative Percent
Valid	Rural	11	15.7	15.7	15.7
	Mixed	52	74.3	74.3	90.0
	Urban	7	10.0	10.0	100.0
	Total	70	100.0	100.0	

Levels of deprivation in area

		Frequency	Percent	Valid Percent	Cumulative Percent
Valid	Predominantly deprived	5	7.1	7.1	7.1
	Mixed	54	77.1	77.1	84.3
	Predominantly affluent	11	15.7	15.7	100.0
	Total	70	100.0	100.0	

Total population served

		Frequency	Percent	Valid Percent	Cumulative Percent
Valid	2000	1	1.4	1.4	1.4
	5000	1	1.4	1.4	2.9
	14000	1	1.4	1.4	4.3
	23500	1	1.4	1.4	5.7
	24000	1	1.4	1.4	7.1
	32000	1	1.4	1.4	8.6
	84500	1	1.4	1.4	10.0
	84544	1	1.4	1.4	11.4
	85000	2	2.9	2.9	14.3
	86000	1	1.4	1.4	15.7
	100000	5	7.1	7.1	22.9
	103500	1	1.4	1.4	24.3
	115000	1	1.4	1.4	25.7
	121000	1	1.4	1.4	27.1
	130000	2	2.9	2.9	30.0
	138000	1	1.4	1.4	31.4
	139000	1	1.4	1.4	32.9
	146700	1	1.4	1.4	34.3

162000	1	1.4	1.4	35.7
165000	2	2.9	2.9	38.6
178000	1	1.4	1.4	40.0
180000	2	2.9	2.9	42.9
188000	1	1.4	1.4	44.3
190000	1	1.4	1.4	45.7
214000	1	1.4	1.4	47.1
240000	1	1.4	1.4	48.6
248000	1	1.4	1.4	50.0
250000	2	2.9	2.9	52.9
260000	1	1.4	1.4	54.3
270000	1	1.4	1.4	55.7
300000	3	4.3	4.3	60.0
320000	1	1.4	1.4	61.4
330000	1	1.4	1.4	62.9
360000	3	4.3	4.3	67.1
375000	2	2.9	2.9	70.0
400000	1	1.4	1.4	71.4
401000	1	1.4	1.4	72.9
450000	1	1.4	1.4	74.3
470000	1	1.4	1.4	75.7
471000	1	1.4	1.4	77.1
500000	3	4.3	4.3	81.4
520000	1	1.4	1.4	82.9
550000	1	1.4	1.4	84.3
610000	1	1.4	1.4	85.7
671000	1	1.4	1.4	87.1
700000	1	1.4	1.4	88.6
731400	1	1.4	1.4	90.0
770000	1	1.4	1.4	91.4

	850000	1	1.4	1.4	92.9
	900000	1	1.4	1.4	94.3
	930000	1	1.4	1.4	95.7
	940000	1	1.4	1.4	97.1
	1000000	1	1.4	1.4	98.6
	1200000	1	1.4	1.4	100.0
	Total	70	100.0	100.0	

Other HaH services operating in same area

		Frequency	Percent	Valid Percent	Cumulative Percent
Valid	Yes	18	25.7	25.7	25.7
	No	52	74.3	74.3	100.0
	Total	70	100.0	100.0	

District Nurse services in area

		Frequency	Percent	Valid Percent	Cumulative Percent
Valid	24h	54	77.1	78.3	78.3
	<24h	15	21.4	21.7	100.0
	Total	69	98.6	100.0	
Missing	System	1	1.4		
Total		70	100.0		

Community specialist palliative care service(s)

		Frequency	Percent	Valid Percent	Cumulative Percent
Valid	Yes	61	87.1	87.1	87.1
	No	9	12.9	12.9	100.0
	Total	70	100.0	100.0	

Marie Curie service

		Frequency	Percent	Valid Percent	Cumulative Percent
Valid	Yes	49	70.0	70.0	70.0
	No	21	30.0	30.0	100.0
	Total	70	100.0	100.0	

Other services in area [OHaH=Other HaH, DN24=24 hour District Nurse, CSPC=Community Specialist Palliative Care, MCur=Marie Curie]

		Frequency	Percent	Valid Percent	Cumulative Percent
Valid	__________CSPC_____	3	4.3	4.3	4.3
	__________CSPC_MCur	8	11.4	11.4	15.7
	_____DN24__________	1	1.4	1.4	17.1
	_____DN24______MCur	2	2.9	2.9	20.0
	_____DN24_CSPC_____	13	18.6	18.6	38.6
	_____DN24_CSPC_MCur	25	35.7	35.7	74.3
	OHaH_______________	1	1.4	1.4	75.7
	OHaH___________MCur	3	4.3	4.3	80.0
	OHaH______CSPC_MCur	1	1.4	1.4	81.4
	OHaH_DN24______MCur	2	2.9	2.9	84.3
	OHaH_DN24_CSPC_____	3	4.3	4.3	88.6
	OHaH_DN24_CSPC_MCur	8	11.4	11.4	100.0
	Total	70	100.0	100.0	

Do patients living in area have access to inpatient palliative care beds

		Frequency	Percent	Valid Percent	Cumulative Percent
Valid	Yes	66	94.3	94.3	94.3

	No	4	5.7	5.7	100.0
	Total	70	100.0	100.0	

Number of Hospice palliative care beds

		Frequency	Percent	Valid Percent	Cumulative Percent
Valid	0	5	7.1	7.5	7.5
	4	1	1.4	1.5	9.0
	6	2	2.9	3.0	11.9
	7	1	1.4	1.5	13.4
	8	3	4.3	4.5	17.9
	9	1	1.4	1.5	19.4
	10	5	7.1	7.5	26.9
	12	7	10.0	10.4	37.3
	13	1	1.4	1.5	38.8
	14	4	5.7	6.0	44.8
	15	3	4.3	4.5	49.3
	16	8	11.4	11.9	61.2
	17	2	2.9	3.0	64.2
	18	7	10.0	10.4	74.6
	19	2	2.9	3.0	77.6
	20	3	4.3	4.5	82.1
	21	1	1.4	1.5	83.6
	23	1	1.4	1.5	85.1
	24	2	2.9	3.0	88.1
	25	3	4.3	4.5	92.5
	30	3	4.3	4.5	97.0
	36	1	1.4	1.5	98.5
	50	1	1.4	1.5	100.0
	Total	67	95.7	100.0	

Missing	System	3	4.3		
Total		70	100.0		

Number of Hospital palliative care beds

		Frequency	Percent	Valid Percent	Cumulative Percent
Valid	0	61	87.1	92.4	92.4
	1	1	1.4	1.5	93.9
	2	1	1.4	1.5	95.5
	3	1	1.4	1.5	97.0
	4	1	1.4	1.5	98.5
	16	1	1.4	1.5	100.0
	Total	66	94.3	100.0	
Missing	System	4	5.7		
Total		70	100.0		

Number of Community Hospital beds

		Frequency	Percent	Valid Percent	Cumulative Percent
Valid	0	49	70.0	74.2	74.2
	1	5	7.1	7.6	81.8
	2	3	4.3	4.5	86.4
	3	3	4.3	4.5	90.9
	4	1	1.4	1.5	92.4
	5	1	1.4	1.5	93.9
	6	3	4.3	4.5	98.5
	15	1	1.4	1.5	100.0
	Total	66	94.3	100.0	
Missing	System	4	5.7		
Total		70	100.0		

Number of Care/Nursing Home beds

		Frequency	Percent	Valid Percent	Cumulative Percent
Valid	0	62	88.6	95.4	95.4
	1	1	1.4	1.5	96.9
	3	1	1.4	1.5	98.5
	10	1	1.4	1.5	100.0
	Total	65	92.9	100.0	
Missing	System	5	7.1		
Total		70	100.0		

Service Referral Criteria: Actively dying - within hours/days

		Frequency	Percent	Valid Percent	Cumulative Percent
Valid	No	10	14.3	14.3	14.3
	Yes	60	85.7	85.7	100.0
	Total	70	100.0	100.0	

Service Referral Criteria: last 2 weeks of life

		Frequency	Percent	Valid Percent	Cumulative Percent
Valid	No	8	11.4	11.4	11.4
	Yes	62	88.6	88.6	100.0
	Total	70	100.0	100.0	

Service Referral Criteria: last month of life

		Frequency	Percent	Valid Percent	Cumulative Percent
Valid	No	17	24.3	24.3	24.3

	Yes	53	75.7	75.7	100.0
	Total	70	100.0	100.0	

Service Referral Criteria: last 3 months of life

		Frequency	Percent	Valid Percent	Cumulative Percent
Valid	No	24	34.3	34.3	34.3
	Yes	46	65.7	65.7	100.0
	Total	70	100.0	100.0	

Service Referral Criteria: last 6 months of life

		Frequency	Percent	Valid Percent	Cumulative Percent
Valid	No	33	47.1	47.1	47.1
	Yes	37	52.9	52.9	100.0
	Total	70	100.0	100.0	

Service Referral Criteria: last year of life

		Frequency	Percent	Valid Percent	Cumulative Percent
Valid	No	34	48.6	48.6	48.6
	Yes	36	51.4	51.4	100.0
	Total	70	100.0	100.0	

Service Referral Criteria: greater than 12 months

		Frequency	Percent	Valid Percent	Cumulative Percent
Valid	No	40	57.1	57.1	57.1
	Yes	30	42.9	42.9	100.0

	Total	70	100.0	100.0	

Service Referral Criteria: Other (specify)

		Frequency	Percent	Valid Percent	Cumulative Percent
Valid		41	58.6	58.6	58.6
	"holding" if capacity eg. to enable discharge from hospital	1	1.4	1.4	60.0
	48 hour palliative care discharge for place of death	1	1.4	1.4	61.4
	Admission avoidance and carer support	1	1.4	1.4	62.9
	Adults patient/ family/ GP consent	1	1.4	1.4	64.3
	all palliative care patients	1	1.4	1.4	65.7
	Bridging service for those expectancy >12 to enable discharge home	1	1.4	1.4	67.1
	Carer breakdown	1	1.4	1.4	68.6
	combination of services	1	1.4	1.4	70.0
	crisis - for hospice	1	1.4	1.4	71.4
	Crisis intervention	1	1.4	1.4	72.9
	Doing all types of referall criteria until CNS joined	1	1.4	1.4	74.3
	greater than 12 months - prioritised	1	1.4	1.4	75.7
	If in crisis - carer breakdown/struggling	1	1.4	1.4	77.1
	If they are on plateau in status they can be reffered greater than 12 months	1	1.4	1.4	78.6
	includes complex wounds	1	1.4	1.4	80.0
	MND cardiac COPD	1	1.4	1.4	81.4

		Frequency	Percent	Valid Percent	Cumulative Percent
	Occasional support for chemo patients	1	1.4	1.4	82.9
	only D/Ns can refer	1	1.4	1.4	84.3
	Partnership for excellance in palliative care (PEPS) criteria used	1	1.4	1.4	85.7
	patients undergoing non-curative treatment	1	1.4	1.4	87.1
	rapid response will go to life expectancy greater 12 months	1	1.4	1.4	88.6
	RC >12 months for certain conditions eg COPD, MND, heart failure	1	1.4	1.4	90.0
	Since Dec 2016 referral reduced	1	1.4	1.4	91.4
	sometimes bridge care - whilst waiting for care package	1	1.4	1.4	92.9
	supporting whilst waiting for admission, hospice discharges	1	1.4	1.4	94.3
	variety	1	1.4	1.4	95.7
	verify expected death	1	1.4	1.4	97.1
	Will step in last 12 months and then refer	1	1.4	1.4	98.6
	worried well anxious public	1	1.4	1.4	100.0
	Total	70	100.0	100.0	

MAXIMUM Life Expectancy referral criteria group

		Frequency	Percent	Valid Percent	Cumulative Percent
Valid	Short-term (Actively dying/Last 2 weeks)	11	15.7	15.7	15.7
	Medium-term (Last month/Last 3 months)	16	22.9	22.9	38.6

	Long-term (6 months to > 1 year)	43	61.4	61.4	100.0
	Total	70	100.0	100.0	

Response time for visit from the service

		Frequency	Percent	Valid Percent	Cumulative Percent
Valid	Within 4 hours	44	62.9	65.7	65.7
	Within 24 hours	20	28.6	29.9	95.5
	Next working day Mon-Fri	3	4.3	4.5	100.0
	Total	67	95.7	100.0	
Missing	System	3	4.3		
Total		70	100.0		

HaH service visits within 4 hours of a Friday 11pm urgent referral

		Frequency	Percent	Valid Percent	Cumulative Percent
Valid	No	23	32.9	34.3	34.3
	Yes	44	62.9	65.7	100.0
	Total	67	95.7	100.0	
Missing	System	3	4.3		
Total		70	100.0		

Service provides: Personal hands on care

		Frequency	Percent	Valid Percent	Cumulative Percent
Valid	Ticked	68	97.1	100.0	100.0
Missing	System	2	2.9		
Total		70	100.0		

When available: Personal hands on care

		Frequency	Percent	Valid Percent	Cumulative Percent
Valid	24/7	35	50.0	52.2	52.2
	8am-8pm, 7 days a week	9	12.9	13.4	65.7
	9am-5pm, 7 days a week	6	8.6	9.0	74.6
	9am-5pm, Mon-Fri	2	2.9	3.0	77.6
	Atypical/other	15	21.4	22.4	100.0
	Total	67	95.7	100.0	
Missing	System	3	4.3		
Total		70	100.0		

Service provides: Hands on care hours is atypical/other description

		Frequency	Percent	Valid Percent	Cumulative Percent
Valid		55	78.6	78.6	78.6
	10pm-7am	1	1.4	1.4	80.0
	10pm-7am 7 days a week	1	1.4	1.4	81.4
	22 hours	1	1.4	1.4	82.9
	7.30am-7.30pm 7 days a week	1	1.4	1.4	84.3
	7.30am-9pm 7 days a week	1	1.4	1.4	85.7
	7am-10pm, 7 days a week	1	1.4	1.4	87.1
	7am-9.30pm 7 days a week	2	2.9	2.9	90.0
	7am-9pm 7 days a week	1	1.4	1.4	91.4
	830AM-10pm 7 days, 24 hr phone	1	1.4	1.4	92.9
	830am-5pm, 10pm-7am 7 days a w	1	1.4	1.4	94.3
	8am-10pm 7 days a week	1	1.4	1.4	95.7
	8am-6pm 7 days a week	1	1.4	1.4	97.1
	9am-5pm or 11pm-7am 7 days	1	1.4	1.4	98.6
	9AM-9PM 7 days a week	1	1.4	1.4	100.0
	Total	70	100.0	100.0	

Service provides: Symptom assessment and management - Physical

		Frequency	Percent	Valid Percent	Cumulative Percent
Valid	Ticked	64	91.4	100.0	100.0
Missing	System	6	8.6		
Total		70	100.0		

When available: Symptom assessment and management - Physical

		Frequency	Percent	Valid Percent	Cumulative Percent
Valid	24/7	39	55.7	60.9	60.9
	8am-8pm, 7 days a week	9	12.9	14.1	75.0
	9am-5pm, 7 days a week	1	1.4	1.6	76.6
	9am-5pm, Mon-Fri	3	4.3	4.7	81.3
	Atypical/other	12	17.1	18.8	100.0
	Total	64	91.4	100.0	
Missing	System	6	8.6		
Total		70	100.0		

Service provides: Symptom assessment and management - Physical - atypical/other description

		Frequency	Percent	Valid Percent	Cumulative Percent
Valid		58	82.9	82.9	82.9
	22 hours	1	1.4	1.4	84.3
	7.30am-7.30pm 7 days a week	1	1.4	1.4	85.7
	7am-10pm, 7 days a week	1	1.4	1.4	87.1
	7am-9.30pm 7 days a week	1	1.4	1.4	88.6
	830am-10pm 7 days	1	1.4	1.4	90.0
	8am-10pm 7 days a week	2	2.9	2.9	92.9

	8am-5pm 7 days a week	1	1.4	1.4	94.3
	8am-6pm 7 days a week	1	1.4	1.4	95.7
	9am-5pm or 11pm-7am 7 days	1	1.4	1.4	97.1
	9AM-9PM 7 days a week	1	1.4	1.4	98.6
	on priority/need	1	1.4	1.4	100.0
	Total	70	100.0	100.0	

HaH service provides 24/7 Clinical care (Hands-on or SAMP)

		Frequency	Percent	Valid Percent	Cumulative Percent
Valid	No	24	34.3	34.3	34.3
	Yes	46	65.7	65.7	100.0
	Total	70	100.0	100.0	

Evidence of 24/7 service [FastR=Fast Response(within 4 hours), HANDS=Hands on care 24/7, SAMP=SAMP 24/7]

		Frequency	Percent	Valid Percent	Cumulative Percent
Valid	________________	16	22.9	22.9	22.9
	____________SAMP	1	1.4	1.4	24.3
	______?????_SAMP	1	1.4	1.4	25.7
	______HANDS_____	1	1.4	1.4	27.1
	______HANDS_SAMP	4	5.7	5.7	32.9
	?????_HANDS_____	1	1.4	1.4	34.3
	?????_HANDS_SAMP	2	2.9	2.9	37.1
	FastR___________	8	11.4	11.4	48.6
	FastR_______SAMP	9	12.9	12.9	61.4
	FastR_HANDS_____	5	7.1	7.1	68.6
	FastR_HANDS_SAMP	22	31.4	31.4	100.0
	Total	70	100.0	100.0	

Service provides: Psychosocial support for Patient and/or Family carers

		Frequency	Percent	Valid Percent	Cumulative Percent
Valid	Ticked	66	94.3	100.0	100.0
Missing	System	4	5.7		
Total		70	100.0		

When available: Psychosocial support for Patient and/or Family carers

		Frequency	Percent	Valid Percent	Cumulative Percent
Valid	24/7	40	57.1	60.6	60.6
	8am-8pm, 7 days a week	6	8.6	9.1	69.7
	9am-5pm, 7 days a week	4	5.7	6.1	75.8
	9am-5pm, Mon-Fri	7	10.0	10.6	86.4
	Atypical/other	9	12.9	13.6	100.0
	Total	66	94.3	100.0	
Missing	System	4	5.7		
Total		70	100.0		

Service provides: Psychosocial care - atypical/other description

		Frequency	Percent	Valid Percent	Cumulative Percent
Valid		60	85.7	85.7	85.7
	22 hours	1	1.4	1.4	87.1
	7.30am-7.30pm 7 days a week	1	1.4	1.4	88.6
	7am-10pm, 7 days a week	1	1.4	1.4	90.0
	7am-9.30pm 7 days a week	1	1.4	1.4	91.4
	830am-10pm 7 days	1	1.4	1.4	92.9
	830am-5pm,10pm-7am 7 days a wk	1	1.4	1.4	94.3
	8am-10pm 7 days a week	1	1.4	1.4	95.7

	8am-6pm 7 days a week	1	1.4	1.4	97.1
	negotiable	1	1.4	1.4	98.6
	on priority/need	1	1.4	1.4	100.0
	Total	70	100.0	100.0	

Service provides: Respite care visits

		Frequency	Percent	Valid Percent	Cumulative Percent
Valid	Ticked	52	74.3	100.0	100.0
Missing	System	18	25.7		
Total		70	100.0		

When available: Respite care visits

		Frequency	Percent	Valid Percent	Cumulative Percent
Valid	Day	15	21.4	29.4	29.4
	Night	2	2.9	3.9	33.3
	Both	33	47.1	64.7	98.0
	Atypical/Other	1	1.4	2.0	100.0
	Total	51	72.9	100.0	
Missing	System	19	27.1		
Total		70	100.0		

Service provides: Respite care - atypical/other description

		Frequency	Percent	Valid Percent	Cumulative Percent
Valid		69	98.6	98.6	98.6
	occasionally	1	1.4	1.4	100.0
	Total	70	100.0	100.0	

Service provides: Practical support for family carers

		Frequency	Percent	Valid Percent	Cumulative Percent
Valid	Ticked	16	22.9	100.0	100.0
Missing	System	54	77.1		
Total		70	100.0		

When available: Practical support for family carers

		Frequency	Percent	Valid Percent	Cumulative Percent
Valid	24/7	2	2.9	13.3	13.3
	8am-8pm, 7 days a week	5	7.1	33.3	46.7
	9am-5pm, 7 days a week	1	1.4	6.7	53.3
	9am-5pm, Mon-Fri	4	5.7	26.7	80.0
	Atypical/other	3	4.3	20.0	100.0
	Total	15	21.4	100.0	
Missing	System	55	78.6		
Total		70	100.0		

Service provides: Practical care - atypical/other description

		Frequency	Percent	Valid Percent	Cumulative Percent
Valid		67	95.7	95.7	95.7
	7am-10pm, 7 days a week	1	1.4	1.4	97.1
	Negotiable	1	1.4	1.4	98.6
	variable - cups of tea etc	1	1.4	1.4	100.0
	Total	70	100.0	100.0	

Service provides: Other (specify)

		Frequency	Percent	Valid Percent	Cumulative Percent
Valid		33	47.1	47.1	47.1
	2 volunteers further respite care	1	1.4	1.4	48.6
	24 hours access from base nurses	1	1.4	1.4	50.0
	Access to distrcit nursing service at night	1	1.4	1.4	51.4
	advance care planning, self-referral, guidelines	1	1.4	1.4	52.9
	can refer to volunteers/care navigation for support	1	1.4	1.4	54.3
	care navigators (volunteers) can stay with patients	1	1.4	1.4	55.7
	Community neighbours scheme, Bereavement services positive living	1	1.4	1.4	57.1
	complimentary therapy	1	1.4	1.4	58.6
	Crisis intervention	1	1.4	1.4	60.0
	Discharge from hospital, sameday care for urgent cases	1	1.4	1.4	61.4
	Enhanced discharge service	1	1.4	1.4	62.9
	Hopsice companions - variable practical support	1	1.4	1.4	64.3
	hospice neighbours - walk dog; hover - appower by volunteers	1	1.4	1.4	65.7
	Hospice neighbours, volunteers	1	1.4	1.4	67.1
	Hospice volunteers	1	1.4	1.4	68.6
	in house agency who provide respite	1	1.4	1.4	70.0
	Light housework, meals, gardening, Night sits 9pm-7am	1	1.4	1.4	71.4
	Macmillan team	1	1.4	1.4	72.9
	Marie Curie provides respite plus volunteers sitting	1	1.4	1.4	74.3

	neighbour networks, cancer care, night sitting	1	1.4	1.4	75.7
	Neighbours and volunteers visit	1	1.4	1.4	77.1
	Pick up drugs in emergency	1	1.4	1.4	78.6
	Provide syringe driver training and EOL care	1	1.4	1.4	80.0
	rapid discharge up to 3 days - all care (some delays)	1	1.4	1.4	81.4
	SAMP/psychosocial - if available staff will provide. Buddy service running too	1	1.4	1.4	82.9
	Specialist lymphoedema , complimentary therapy, bereavement support	1	1.4	1.4	84.3
	support for CSCI use in Nursing Home	1	1.4	1.4	85.7
	verification of death, integrated team	1	1.4	1.4	87.1
	Volunteer companions	1	1.4	1.4	88.6
	Volunteer sector	1	1.4	1.4	90.0
	volunteer services	2	2.9	2.9	92.9
	Volunteer sitting service	1	1.4	1.4	94.3
	volunteers - carer companions; for patients and carers in areas with no DN - H@H cover for pal care	1	1.4	1.4	95.7
	Volunteers can help	1	1.4	1.4	97.1
	Volunteers offer companionship and support	1	1.4	1.4	98.6
	Volunteers, family councilling support and complimentary therapist	1	1.4	1.4	100.0
	Total	70	100.0	100.0	

HaH service provides Abundant Supportive care (2+ from Psychosocial/Respite/Practical) for family carers

		Frequency	Percent	Valid Percent	Cumulative Percent
Valid	No	15	21.4	21.4	21.4
	Yes	55	78.6	78.6	100.0
	Total	70	100.0	100.0	

Number of Referrals to the service

		Frequency	Percent	Valid Percent	Cumulative Percent
Valid	62	1	1.4	1.6	1.6
	83	1	1.4	1.6	3.2
	87	1	1.4	1.6	4.8
	100	4	5.7	6.5	11.3
	107	1	1.4	1.6	12.9
	111	1	1.4	1.6	14.5
	112	1	1.4	1.6	16.1
	113	1	1.4	1.6	17.7
	152	1	1.4	1.6	19.4
	167	1	1.4	1.6	21.0
	175	1	1.4	1.6	22.6
	190	1	1.4	1.6	24.2
	197	1	1.4	1.6	25.8
	200	3	4.3	4.8	30.6
	232	1	1.4	1.6	32.3
	233	1	1.4	1.6	33.9
	235	1	1.4	1.6	35.5
	240	1	1.4	1.6	37.1
	270	1	1.4	1.6	38.7

	286	1	1.4	1.6	40.3
	298	1	1.4	1.6	41.9
	304	1	1.4	1.6	43.5
	323	1	1.4	1.6	45.2
	329	1	1.4	1.6	46.8
	353	1	1.4	1.6	48.4
	360	1	1.4	1.6	50.0
	362	1	1.4	1.6	51.6
	380	1	1.4	1.6	53.2
	388	1	1.4	1.6	54.8
	420	3	4.3	4.8	59.7
	425	1	1.4	1.6	61.3
	427	1	1.4	1.6	62.9
	442	1	1.4	1.6	64.5
	450	1	1.4	1.6	66.1
	498	1	1.4	1.6	67.7
	499	1	1.4	1.6	69.4
	516	1	1.4	1.6	71.0
	519	1	1.4	1.6	72.6
	547	1	1.4	1.6	74.2
	600	2	2.9	3.2	77.4
	635	1	1.4	1.6	79.0
	640	1	1.4	1.6	80.6
	685	1	1.4	1.6	82.3
	693	1	1.4	1.6	83.9
	720	2	2.9	3.2	87.1
	782	1	1.4	1.6	88.7
	803	1	1.4	1.6	90.3
	890	1	1.4	1.6	91.9
	1173	1	1.4	1.6	93.5

	1180	1	1.4	1.6	95.2
	1470	1	1.4	1.6	96.8
	1500	1	1.4	1.6	98.4
	2222	1	1.4	1.6	100.0
	Total	62	88.6	100.0	
Missing	System	8	11.4		
Total		70	100.0		

Number of referrals group

		Frequency	Percent	Valid Percent	Cumulative Percent
Valid	Low (up to 200)	19	27.1	30.6	30.6
	Medium (201-500)	24	34.3	38.7	69.4
	High (over 500)	19	27.1	30.6	100.0
	Total	62	88.6	100.0	
Missing	System	8	11.4		
Total		70	100.0		

Duration of service use for most patients

		Frequency	Percent	Valid Percent	Cumulative Percent
Valid	< 1 week	9	12.9	15.0	15.0
	1 week - 2 months	36	51.4	60.0	75.0
	> 2 months	15	21.4	25.0	100.0
	Total	60	85.7	100.0	
Missing	System	10	14.3		
Total		70	100.0		

Intensity of service for most patients

		Frequency	Percent	Valid Percent	Cumulative Percent
Valid	< 3h care/week	2	2.9	3.1	3.1
	Between 3h care/day - 3h care/week	16	22.9	25.0	28.1
	> 3h care/day	32	45.7	50.0	78.1
	A roughly even split of the above	14	20.0	21.9	100.0
	Total	64	91.4	100.0	
Missing	System	6	8.6		
Total		70	100.0		

Intensity of care > 3 hours per day

		Frequency	Percent	Valid Percent	Cumulative Percent
Valid	No	32	45.7	50.0	50.0
	Yes	32	45.7	50.0	100.0
	Total	64	91.4	100.0	
Missing	System	6	8.6		
Total		70	100.0		

Number of Staff: Healthcare assistants

		Frequency	Percent	Valid Percent	Cumulative Percent
Valid	0	9	12.9	13.2	13.2
	1	1	1.4	1.5	14.7
	2	2	2.9	2.9	17.6
	3	3	4.3	4.4	22.1
	4	7	10.0	10.3	32.4
	5	2	2.9	2.9	35.3
	6	3	4.3	4.4	39.7
	7	8	11.4	11.8	51.5
	8	9	12.9	13.2	64.7

	9	5	7.1	7.4	72.1
	10	2	2.9	2.9	75.0
	12	2	2.9	2.9	77.9
	13	4	5.7	5.9	83.8
	15	1	1.4	1.5	85.3
	16	2	2.9	2.9	88.2
	17	1	1.4	1.5	89.7
	18	2	2.9	2.9	92.6
	21	2	2.9	2.9	95.6
	26	1	1.4	1.5	97.1
	37	1	1.4	1.5	98.5
	40	1	1.4	1.5	100.0
	Total	68	97.1	100.0	
Missing	System	2	2.9		
Total		70	100.0		

Whole Time Equivalent(WTE) staff,if not all full-time: Healthcare assistants

		Frequency	Percent	Valid Percent	Cumulative Percent
Valid	.00	5	7.1	7.4	7.4
	.60	1	1.4	1.5	8.8
	.70	1	1.4	1.5	10.3
	1.40	1	1.4	1.5	11.8
	2.00	2	2.9	2.9	14.7
	2.40	1	1.4	1.5	16.2
	2.60	1	1.4	1.5	17.6
	2.80	2	2.9	2.9	20.6
	3.00	4	5.7	5.9	26.5
	3.16	1	1.4	1.5	27.9
	3.17	1	1.4	1.5	29.4

	3.50	1	1.4	1.5	30.9
	3.60	1	1.4	1.5	32.4
	3.73	1	1.4	1.5	33.8
	4.00	6	8.6	8.8	42.6
	4.44	1	1.4	1.5	44.1
	5.00	2	2.9	2.9	47.1
	5.25	1	1.4	1.5	48.5
	5.30	1	1.4	1.5	50.0
	5.60	1	1.4	1.5	51.5
	5.68	1	1.4	1.5	52.9
	6.00	4	5.7	5.9	58.8
	6.20	1	1.4	1.5	60.3
	7.40	1	1.4	1.5	61.8
	7.41	1	1.4	1.5	63.2
	7.50	1	1.4	1.5	64.7
	7.53	1	1.4	1.5	66.2
	7.54	1	1.4	1.5	67.6
	8.00	4	5.7	5.9	73.5
	8.20	1	1.4	1.5	75.0
	8.80	1	1.4	1.5	76.5
	9.50	1	1.4	1.5	77.9
	9.60	1	1.4	1.5	79.4
	10.25	1	1.4	1.5	80.9
	10.33	1	1.4	1.5	82.4
	11.61	1	1.4	1.5	83.8
	11.80	1	1.4	1.5	85.3
	12.00	2	2.9	2.9	88.2
	13.00	1	1.4	1.5	89.7
	14.00	2	2.9	2.9	92.6
	15.75	1	1.4	1.5	94.1

	16.00	1	1.4	1.5	95.6
	16.30	1	1.4	1.5	97.1
	16.36	1	1.4	1.5	98.5
	20.50	1	1.4	1.5	100.0
	Total	68	97.1	100.0	
Missing	System	2	2.9		
Total		70	100.0		

Number of Staff: Registered Nurses

		Frequency	Percent	Valid Percent	Cumulative Percent
Valid	0	10	14.3	14.7	14.7
	1	9	12.9	13.2	27.9
	2	6	8.6	8.8	36.8
	3	1	1.4	1.5	38.2
	4	8	11.4	11.8	50.0
	5	4	5.7	5.9	55.9
	6	7	10.0	10.3	66.2
	7	6	8.6	8.8	75.0
	8	4	5.7	5.9	80.9
	9	3	4.3	4.4	85.3
	10	2	2.9	2.9	88.2
	12	2	2.9	2.9	91.2
	13	3	4.3	4.4	95.6
	18	1	1.4	1.5	97.1
	19	1	1.4	1.5	98.5
	22	1	1.4	1.5	100.0
	Total	68	97.1	100.0	
Missing	System	2	2.9		
Total		70	100.0		

Whole Time Equivalent(WTE) staff,if not all full-time: Registered Nurses

		Frequency	Percent	Valid Percent	Cumulative Percent
Valid	.00	7	10.0	10.3	10.3
	.50	1	1.4	1.5	11.8
	.75	2	2.9	2.9	14.7
	.80	3	4.3	4.4	19.1
	.90	1	1.4	1.5	20.6
	.93	1	1.4	1.5	22.1
	1.00	4	5.7	5.9	27.9
	1.13	1	1.4	1.5	29.4
	1.20	1	1.4	1.5	30.9
	1.50	1	1.4	1.5	32.4
	2.00	2	2.9	2.9	35.3
	2.40	1	1.4	1.5	36.8
	2.60	2	2.9	2.9	39.7
	2.80	1	1.4	1.5	41.2
	2.89	1	1.4	1.5	42.6
	3.00	1	1.4	1.5	44.1
	3.20	2	2.9	2.9	47.1
	3.60	2	2.9	2.9	50.0
	3.73	1	1.4	1.5	51.5
	3.80	1	1.4	1.5	52.9
	4.00	6	8.6	8.8	61.8
	4.33	1	1.4	1.5	63.2
	4.40	1	1.4	1.5	64.7
	4.50	2	2.9	2.9	67.6
	4.80	2	2.9	2.9	70.6
	5.10	1	1.4	1.5	72.1

	5.25	2	2.9	2.9	75.0
	5.50	1	1.4	1.5	76.5
	5.63	1	1.4	1.5	77.9
	6.00	2	2.9	2.9	80.9
	6.50	1	1.4	1.5	82.4
	6.75	1	1.4	1.5	83.8
	7.00	2	2.9	2.9	86.8
	8.00	2	2.9	2.9	89.7
	8.20	1	1.4	1.5	91.2
	8.33	1	1.4	1.5	92.6
	8.40	1	1.4	1.5	94.1
	10.00	1	1.4	1.5	95.6
	11.00	1	1.4	1.5	97.1
	16.20	1	1.4	1.5	98.5
	16.50	1	1.4	1.5	100.0
	Total	68	97.1	100.0	
Missing	System	2	2.9		
Total		70	100.0		

Number of Staff: Medical Consultants

		Frequency	Percent	Valid Percent	Cumulative Percent
Valid	0	50	71.4	73.5	73.5
	1	14	20.0	20.6	94.1
	2	2	2.9	2.9	97.1
	3	2	2.9	2.9	100.0
	Total	68	97.1	100.0	
Missing	System	2	2.9		
Total		70	100.0		

Whole Time Equivalent(WTE) staff,if not all full-time: Medical Consultants

		Frequency	Percent	Valid Percent	Cumulative Percent
Valid	.00	49	70.0	72.1	72.1
	.20	2	2.9	2.9	75.0
	.30	1	1.4	1.5	76.5
	.50	1	1.4	1.5	77.9
	.60	4	5.7	5.9	83.8
	.75	7	10.0	10.3	94.1
	1.00	1	1.4	1.5	95.6
	1.40	1	1.4	1.5	97.1
	1.50	2	2.9	2.9	100.0
	Total	68	97.1	100.0	
Missing	System	2	2.9		
Total		70	100.0		

Number of Staff: Other Doctors

		Frequency	Percent	Valid Percent	Cumulative Percent
Valid	0	51	72.9	75.0	75.0
	1	13	18.6	19.1	94.1
	2	3	4.3	4.4	98.5
	3	1	1.4	1.5	100.0
	Total	68	97.1	100.0	
Missing	System	2	2.9		
Total		70	100.0		

Whole Time Equivalent(WTE) staff,if not all full-time: Other Doctors

	Frequency	Percent	Valid Percent	Cumulative Percent

Valid	.00	50	71.4	73.5	73.5
	.10	1	1.4	1.5	75.0
	.20	2	2.9	2.9	77.9
	.50	9	12.9	13.2	91.2
	.80	3	4.3	4.4	95.6
	1.00	2	2.9	2.9	98.5
	2.00	1	1.4	1.5	100.0
	Total	68	97.1	100.0	
Missing	System	2	2.9		
Total		70	100.0		

Number of Staff: Physiotherapists

		Frequency	Percent	Valid Percent	Cumulative Percent
Valid	0	51	72.9	75.0	75.0
	1	15	21.4	22.1	97.1
	2	2	2.9	2.9	100.0
	Total	68	97.1	100.0	
Missing	System	2	2.9		
Total		70	100.0		

Whole Time Equivalent(WTE) staff,if not all full-time: Physiotherapists

		Frequency	Percent	Valid Percent	Cumulative Percent
Valid	.00	51	72.9	75.0	75.0
	.50	9	12.9	13.2	88.2
	.60	2	2.9	2.9	91.2
	.80	2	2.9	2.9	94.1
	1.00	3	4.3	4.4	98.5
	1.20	1	1.4	1.5	100.0

	Total	68	97.1	100.0	
Missing	System	2	2.9		
Total		70	100.0		

Number of Staff: Occupational Therapists

		Frequency	Percent	Valid Percent	Cumulative Percent
Valid	0	53	75.7	77.9	77.9
	1	14	20.0	20.6	98.5
	2	1	1.4	1.5	100.0
	Total	68	97.1	100.0	
Missing	System	2	2.9		
Total		70	100.0		

Whole Time Equivalent(WTE) staff,if not all full-time: Occupational Therapists

		Frequency	Percent	Valid Percent	Cumulative Percent
Valid	.00	52	74.3	76.5	76.5
	.50	9	12.9	13.2	89.7
	.60	1	1.4	1.5	91.2
	.80	1	1.4	1.5	92.6
	1.00	4	5.7	5.9	98.5
	1.40	1	1.4	1.5	100.0
	Total	68	97.1	100.0	
Missing	System	2	2.9		
Total		70	100.0		

Number of Staff: Counselling

		Frequency	Percent	Valid Percent	Cumulative Percent

Valid	0	46	65.7	67.6	67.6
	1	14	20.0	20.6	88.2
	2	2	2.9	2.9	91.2
	3	5	7.1	7.4	98.5
	4	1	1.4	1.5	100.0
	Total	68	97.1	100.0	
Missing	System	2	2.9		
Total		70	100.0		

Whole Time Equivalent(WTE) staff,if not all full-time: Counselling

		Frequency	Percent	Valid Percent	Cumulative Percent
Valid	.00	46	65.7	67.6	67.6
	.50	10	14.3	14.7	82.4
	.64	1	1.4	1.5	83.8
	.80	1	1.4	1.5	85.3
	1.00	3	4.3	4.4	89.7
	1.50	1	1.4	1.5	91.2
	1.80	1	1.4	1.5	92.6
	2.00	2	2.9	2.9	95.6
	2.20	1	1.4	1.5	97.1
	2.50	1	1.4	1.5	98.5
	3.00	1	1.4	1.5	100.0
	Total	68	97.1	100.0	
Missing	System	2	2.9		
Total		70	100.0		

Number of Staff: Social work

		Frequency	Percent	Valid Percent	Cumulative Percent

Valid	0	59	84.3	86.8	86.8
	1	8	11.4	11.8	98.5
	2	1	1.4	1.5	100.0
	Total	68	97.1	100.0	
Missing	System	2	2.9		
Total		70	100.0		

Whole Time Equivalent(WTE) staff,if not all full-time: Social work

		Frequency	Percent	Valid Percent	Cumulative Percent
Valid	.00	59	84.3	86.8	86.8
	.50	6	8.6	8.8	95.6
	.90	1	1.4	1.5	97.1
	1.00	2	2.9	2.9	100.0
	Total	68	97.1	100.0	
Missing	System	2	2.9		
Total		70	100.0		

Number of Staff: Chaplaincy

		Frequency	Percent	Valid Percent	Cumulative Percent
Valid	0	53	75.7	77.9	77.9
	1	14	20.0	20.6	98.5
	2	1	1.4	1.5	100.0
	Total	68	97.1	100.0	
Missing	System	2	2.9		
Total		70	100.0		

Whole Time Equivalent(WTE) staff,if not all full-time: Chaplaincy

		Frequency	Percent	Valid Percent	Cumulative Percent
Valid	.00	52	74.3	76.5	76.5
	.20	1	1.4	1.5	77.9
	.26	1	1.4	1.5	79.4
	.50	11	15.7	16.2	95.6
	.60	1	1.4	1.5	97.1
	1.00	2	2.9	2.9	100.0
	Total	68	97.1	100.0	
Missing	System	2	2.9		
Total		70	100.0		

Number of Staff: Volunteers

		Frequency	Percent	Valid Percent	Cumulative Percent
Valid	0	42	60.0	61.8	61.8
	1	4	5.7	5.9	67.6
	2	4	5.7	5.9	73.5
	5	2	2.9	2.9	76.5
	6	2	2.9	2.9	79.4
	8	1	1.4	1.5	80.9
	9	1	1.4	1.5	82.4
	10	1	1.4	1.5	83.8
	15	4	5.7	5.9	89.7
	19	1	1.4	1.5	91.2
	20	1	1.4	1.5	92.6
	21	1	1.4	1.5	94.1
	37	1	1.4	1.5	95.6
	52	1	1.4	1.5	97.1
	108	1	1.4	1.5	98.5
	220	1	1.4	1.5	100.0

	Total	68	97.1	100.0	
Missing	System	2	2.9		
Total		70	100.0		

Whole Time Equivalent(WTE) staff,if not all full-time: Volunteers

		Frequency	Percent	Valid Percent	Cumulative Percent
Valid	.00	42	60.0	61.8	61.8
	.20	3	4.3	4.4	66.2
	.40	4	5.7	5.9	72.1
	.50	1	1.4	1.5	73.5
	1.00	2	2.9	2.9	76.5
	1.20	2	2.9	2.9	79.4
	1.60	1	1.4	1.5	80.9
	1.80	1	1.4	1.5	82.4
	2.00	1	1.4	1.5	83.8
	3.00	4	5.7	5.9	89.7
	3.80	1	1.4	1.5	91.2
	4.00	1	1.4	1.5	92.6
	4.20	1	1.4	1.5	94.1
	7.40	1	1.4	1.5	95.6
	10.40	1	1.4	1.5	97.1
	21.60	1	1.4	1.5	98.5
	44.00	1	1.4	1.5	100.0
	Total	68	97.1	100.0	
Missing	System	2	2.9		
Total		70	100.0		

Number of Staff: Administrative

		Frequency	Percent	Valid Percent	Cumulative Percent
Valid	0	21	30.0	30.9	30.9
	1	30	42.9	44.1	75.0
	2	10	14.3	14.7	89.7
	3	2	2.9	2.9	92.6
	4	2	2.9	2.9	95.6
	5	3	4.3	4.4	100.0
	Total	68	97.1	100.0	
Missing	System	2	2.9		
Total		70	100.0		

Whole Time Equivalent(WTE) staff,if not all full-time: Administrative

		Frequency	Percent	Valid Percent	Cumulative Percent
Valid	.00	20	28.6	29.4	29.4
	.20	1	1.4	1.5	30.9
	.50	2	2.9	2.9	33.8
	.53	1	1.4	1.5	35.3
	.60	3	4.3	4.4	39.7
	.80	3	4.3	4.4	44.1
	.90	5	7.1	7.4	51.5
	1.00	18	25.7	26.5	77.9
	1.20	1	1.4	1.5	79.4
	1.60	2	2.9	2.9	82.4
	1.75	1	1.4	1.5	83.8
	1.80	4	5.7	5.9	89.7
	2.00	1	1.4	1.5	91.2
	3.00	1	1.4	1.5	92.6
	3.30	1	1.4	1.5	94.1
	4.00	2	2.9	2.9	97.1

		Frequency	Percent	Valid Percent	Cumulative Percent
	4.20	1	1.4	1.5	98.5
	5.00	1	1.4	1.5	100.0
	Total	68	97.1	100.0	
Missing	System	2	2.9		
Total		70	100.0		

Number of Staff: Management

		Frequency	Percent	Valid Percent	Cumulative Percent
Valid	0	7	10.0	10.3	10.3
	1	52	74.3	76.5	86.8
	2	5	7.1	7.4	94.1
	3	3	4.3	4.4	98.5
	4	1	1.4	1.5	100.0
	Total	68	97.1	100.0	
Missing	System	2	2.9		
Total		70	100.0		

Whole Time Equivalent(WTE) staff,if not all full-time: Management

		Frequency	Percent	Valid Percent	Cumulative Percent
Valid	.00	7	10.0	10.3	10.3
	.30	2	2.9	2.9	13.2
	.40	1	1.4	1.5	14.7
	.50	2	2.9	2.9	17.6
	.58	1	1.4	1.5	19.1
	.80	8	11.4	11.8	30.9
	.85	1	1.4	1.5	32.4
	1.00	39	55.7	57.4	89.7
	2.00	5	7.1	7.4	97.1

	3.00	1	1.4	1.5	98.5
	4.00	1	1.4	1.5	100.0
	Total	68	97.1	100.0	
Missing	System	2	2.9		
Total		70	100.0		

Service staff: Other (specify)

		Frequency	Percent	Valid Percent	Cumulative Percent
Valid		33	47.1	47.1	47.1
	21 nurses are bank nurses, also have nurse co-ordinators, trustee doctors and consultants	1	1.4	1.4	48.6
	3 care co-ordinators	1	1.4	1.4	50.0
	Access to staff where FTE not specified, lots of volunteers	1	1.4	1.4	51.4
	advanced nurse practitioner	1	1.4	1.4	52.9
	All other staff apart HCA RN available through PEPS	1	1.4	1.4	54.3
	assistant practitioner	1	1.4	1.4	55.7
	Assistant practitioners, occupational therapy team, lymphodaema team + vacancy	1	1.4	1.4	57.1
	Associate director, chief executive	1	1.4	1.4	58.6
	Bank for HCAs	1	1.4	1.4	60.0
	CEO, lymphodoema team, complementary therapist	1	1.4	1.4	61.4
	clinical admin team	1	1.4	1.4	62.9
	Clinical Nurse Specialist CLinical Manager	1	1.4	1.4	64.3
	Clinical nurse specialists	2	2.9	2.9	67.1

	clinical service	1	1.4	1.4	68.6
	co-ordinator	1	1.4	1.4	70.0
	co-ordinators	1	1.4	1.4	71.4
	community manager	1	1.4	1.4	72.9
	Complementary therapist	1	1.4	1.4	74.3
	Complimentary therapy available	1	1.4	1.4	75.7
	Deputy manager	1	1.4	1.4	77.1
	deputy of care	1	1.4	1.4	78.6
	Deputy Team Lead	1	1.4	1.4	80.0
	Hub Band 7; Therapy Lead	1	1.4	1.4	81.4
	large team of counsellors and chaplaincy staff	1	1.4	1.4	82.9
	Macmillan QOL patient; children's bereavement	1	1.4	1.4	84.3
	Management provided by nurses	1	1.4	1.4	85.7
	No individual figures as inpatient unit + H@H is integrated. Big NHS Trust.	1	1.4	1.4	87.1
	nurse co-ordinator	1	1.4	1.4	88.6
	Occupational therapist, physio, counselling and chaplaincy available not dedicated	1	1.4	1.4	90.0
	Paramedic	1	1.4	1.4	91.4
	PEP director	1	1.4	1.4	92.9
	referal co-ordinator	1	1.4	1.4	94.3
	Team leader	1	1.4	1.4	95.7
	trained nurse and co-ordinator	1	1.4	1.4	97.1
	Use mix of bank staff and trained staff. Benefits office advice	1	1.4	1.4	98.6
	Volunteer listeners	1	1.4	1.4	100.0
	Total	70	100.0	100.0	

Number of Staff: Other Service

		Frequency	Percent	Valid Percent	Cumulative Percent
Valid	0	45	64.3	66.2	66.2
	1	12	17.1	17.6	83.8
	2	4	5.7	5.9	89.7
	3	2	2.9	2.9	92.6
	4	2	2.9	2.9	95.6
	5	1	1.4	1.5	97.1
	13	1	1.4	1.5	98.5
	25	1	1.4	1.5	100.0
	Total	68	97.1	100.0	
Missing	System	2	2.9		
Total		70	100.0		

Whole Time Equivalent(WTE) staff,if not all full-time: Other Service

		Frequency	Percent	Valid Percent	Cumulative Percent
Valid	.00	44	62.9	64.7	64.7
	.33	1	1.4	1.5	66.2
	.40	2	2.9	2.9	69.1
	.60	1	1.4	1.5	70.6
	.75	4	5.7	5.9	76.5
	.80	1	1.4	1.5	77.9
	1.00	3	4.3	4.4	82.4
	1.40	1	1.4	1.5	83.8
	1.50	4	5.7	5.9	89.7
	1.60	2	2.9	2.9	92.6
	2.80	1	1.4	1.5	94.1
	3.04	1	1.4	1.5	95.6

	3.75	1	1.4	1.5	97.1
	5.00	1	1.4	1.5	98.5
	9.75	1	1.4	1.5	100.0
	Total	68	97.1	100.0	
Missing	System	2	2.9		
Total		70	100.0		

Staff [HA=Healthcare Assistants, RN=Registered Nurses, DR=Medical consultant/Other doctor, TC=Physio/OT/Counselling, SV=Social work/Chaplaincy/Volunteers]

		Frequency	Percent	Valid Percent	Cumulative Percent
Valid	______DR_TC_SV	1	1.4	1.4	1.4
	___RN_________	1	1.4	1.4	2.9
	___RN_______SV	1	1.4	1.4	4.3
	___RN_DR______	1	1.4	1.4	5.7
	___RN_DR_TC_SV	1	1.4	1.4	7.1
	??_??_??_??_??	2	2.9	2.9	10.0
	HA____________	2	2.9	2.9	12.9
	HA__________SV	1	1.4	1.4	14.3
	HA_______TC___	1	1.4	1.4	15.7
	HA_______TC_SV	1	1.4	1.4	17.1
	HA____DR______	1	1.4	1.4	18.6
	HA_RN_________	23	32.9	32.9	51.4
	HA_RN_______SV	8	11.4	11.4	62.9
	HA_RN____TC_SV	7	10.0	10.0	72.9
	HA_RN_DR______	5	7.1	7.1	80.0
	HA_RN_DR____SV	1	1.4	1.4	81.4
	HA_RN_DR_TC___	1	1.4	1.4	82.9
	HA_RN_DR_TC_SV	12	17.1	17.1	100.0
	Total	70	100.0	100.0	

SUPPORT for service from: Local Commissioners

		Frequency	Percent	Valid Percent	Cumulative Percent
Valid	Not at all	8	11.4	11.4	11.4
	Somewhat	32	45.7	45.7	57.1
	Substantially	30	42.9	42.9	100.0
	Total	70	100.0	100.0	

SUPPORT for service from: Board of Trustees of charity

		Frequency	Percent	Valid Percent	Cumulative Percent
Valid	Somewhat	6	8.6	8.6	8.6
	Substantially	64	91.4	91.4	100.0
	Total	70	100.0	100.0	

SUPPORT for service from: Local Hospice

		Frequency	Percent	Valid Percent	Cumulative Percent
Valid	Somewhat	4	5.7	5.8	5.8
	Substantially	65	92.9	94.2	100.0
	Total	69	98.6	100.0	
Missing	System	1	1.4		
Total		70	100.0		

SUPPORT for service from: Local generic Community Nursing services

		Frequency	Percent	Valid Percent	Cumulative Percent
Valid	Somewhat	22	31.4	31.4	31.4
	Substantially	48	68.6	68.6	100.0

	Total	70	100.0	100.0	

SUPPORT for service from: Local GPs

		Frequency	Percent	Valid Percent	Cumulative Percent
Valid	Somewhat	32	45.7	45.7	45.7
	Substantially	38	54.3	54.3	100.0
	Total	70	100.0	100.0	

SUPPORT for service from: Other local service (specify)

		Frequency	Percent	Valid Percent	Cumulative Percent
Valid		12	17.1	17.1	17.1
	acute clinical nurse specialist	1	1.4	1.4	18.6
	Care agencies	2	2.9	2.9	21.4
	Care home team, education and support, neurology team, community Macmillan	1	1.4	1.4	22.9
	chaplain/spiritual leader volunteer	1	1.4	1.4	24.3
	Clinical Nurse Specialist from hospice community team	1	1.4	1.4	25.7
	Clinical Nurse Specialist in hospital	1	1.4	1.4	27.1
	Clinical Nurse specialists - hospital	1	1.4	1.4	28.6
	CNS hospital + hospice	1	1.4	1.4	30.0
	community Macmillan	1	1.4	1.4	31.4
	Community Multi-disciplinary team	1	1.4	1.4	32.9
	continual health funding	1	1.4	1.4	34.3
	continuing health funding	1	1.4	1.4	35.7
	continuing healthcare; REWCS; assessment direct	1	1.4	1.4	37.1

	District nurses	1	1.4	1.4	38.6
	ECHO team	1	1.4	1.4	40.0
	Health funding	1	1.4	1.4	41.4
	hospital Macmillan and CHC teams	1	1.4	1.4	42.9
	hospital services, local community rapid response,	1	1.4	1.4	44.3
	league of friends	1	1.4	1.4	45.7
	local care home and pharmicies	1	1.4	1.4	47.1
	Local commissioning groups	1	1.4	1.4	48.6
	Macmillan	4	5.7	5.7	54.3
	Macmillan benefits advice office	1	1.4	1.4	55.7
	Macmillan services	1	1.4	1.4	57.1
	Macmillan support service	1	1.4	1.4	58.6
	Macmillan team - MDT meetings	1	1.4	1.4	60.0
	Marie Curie	7	10.0	10.0	70.0
	Multi-faith chaplains	1	1.4	1.4	71.4
	Muslim burial service, chaplaincy, ICRS, Marie Curie	1	1.4	1.4	72.9
	Neuroteam	1	1.4	1.4	74.3
	Numerous other	1	1.4	1.4	75.7
	Partnership between hospital and hospice	1	1.4	1.4	77.1
	Partnership for excellence in palliative care	1	1.4	1.4	78.6
	Private agency	1	1.4	1.4	80.0
	Professions allied to medicines	1	1.4	1.4	81.4
	Royal United Hospital	1	1.4	1.4	82.9
	Social services	12	17.1	17.1	100.0
	Total	70	100.0	100.0	

SUPPORT for service from: Other local service

		Frequency	Percent	Valid Percent	Cumulative Percent
Valid	Not at all	3	4.3	5.1	5.1
	Somewhat	22	31.4	37.3	42.4
	Substantially	34	48.6	57.6	100.0
	Total	59	84.3	100.0	
Missing	System	11	15.7		
Total		70	100.0		

SUPPORT for service via: Manageable number of referrals

		Frequency	Percent	Valid Percent	Cumulative Percent
Valid	Not at all	2	2.9	2.9	2.9
	Somewhat	26	37.1	37.1	40.0
	Substantially	42	60.0	60.0	100.0
	Total	70	100.0	100.0	

SUPPORT for service via: Suitable/appropriate referrals

		Frequency	Percent	Valid Percent	Cumulative Percent
Valid	Not at all	2	2.9	2.9	2.9
	Somewhat	25	35.7	35.7	38.6
	Substantially	43	61.4	61.4	100.0
	Total	70	100.0	100.0	

SUPPORT for service via: Adequate funding

		Frequency	Percent	Valid Percent	Cumulative Percent
Valid	Not at all	9	12.9	12.9	12.9

		Frequency	Percent	Valid Percent	Cumulative Percent
	Somewhat	37	52.9	52.9	65.7
	Substantially	24	34.3	34.3	100.0
	Total	70	100.0	100.0	

SUPPORT for service via: Ability to recruit and retain suitable staff

		Frequency	Percent	Valid Percent	Cumulative Percent
Valid	Not at all	1	1.4	1.4	1.4
	Somewhat	27	38.6	38.6	40.0
	Substantially	42	60.0	60.0	100.0
	Total	70	100.0	100.0	

SUPPORT for service via: Other services (e.g. social services)

		Frequency	Percent	Valid Percent	Cumulative Percent
Valid	Not at all	14	20.0	20.3	20.3
	Somewhat	45	64.3	65.2	85.5
	Substantially	10	14.3	14.5	100.0
	Total	69	98.6	100.0	
Missing	System	1	1.4		
Total		70	100.0		

SUPPORT for service via: Ability to provide Out Of Hours

		Frequency	Percent	Valid Percent	Cumulative Percent
Valid	Not at all	2	2.9	2.9	2.9
	Somewhat	23	32.9	33.3	36.2
	Substantially	44	62.9	63.8	100.0
	Total	69	98.6	100.0	
Missing	System	1	1.4		

Total	70	100.0		

SUPPORT for service via: Ability to access necessary clinical equipment in a timely fashion

		Frequency	Percent	Valid Percent	Cumulative Percent
Valid	Not at all	3	4.3	4.3	4.3
	Somewhat	31	44.3	44.3	48.6
	Substantially	36	51.4	51.4	100.0
	Total	70	100.0	100.0	

SUPPORT for service via: Ability to access anticipatory medications by injection in a timely fashion

		Frequency	Percent	Valid Percent	Cumulative Percent
Valid	Not at all	2	2.9	2.9	2.9
	Somewhat	29	41.4	41.4	44.3
	Substantially	39	55.7	55.7	100.0
	Total	70	100.0	100.0	

SUPPORT for service via: Ability to provide administration of anticipatory medications by injection when needed

		Frequency	Percent	Valid Percent	Cumulative Percent
Valid	Not at all	2	2.9	2.9	2.9
	Somewhat	40	57.1	57.1	60.0
	Substantially	28	40.0	40.0	100.0
	Total	70	100.0	100.0	

SUPPORT for service via: Geography of area (e.g. distances,parking,traffic,safety)

		Frequency	Percent	Valid Percent	Cumulative Percent

Valid	Not at all	3	4.3	4.3	4.3
	Somewhat	52	74.3	74.3	78.6
	Substantially	15	21.4	21.4	100.0
	Total	70	100.0	100.0	

Make it DIFFICULT running service: Lack of support from Local Commissioners

		Frequency	Percent	Valid Percent	Cumulative Percent
Valid	Not at all	32	45.7	46.4	46.4
	Somewhat	28	40.0	40.6	87.0
	Substantially	9	12.9	13.0	100.0
	Total	69	98.6	100.0	
Missing	System	1	1.4		
Total		70	100.0		

Make it DIFFICULT running service: Lack of support from Board of Trustees of charity

		Frequency	Percent	Valid Percent	Cumulative Percent
Valid	Not at all	62	88.6	88.6	88.6
	Somewhat	3	4.3	4.3	92.9
	Substantially	5	7.1	7.1	100.0
	Total	70	100.0	100.0	

Make it DIFFICULT running service: Relationship with Local Hospice

		Frequency	Percent	Valid Percent	Cumulative Percent
Valid	Not at all	62	88.6	89.9	89.9
	Somewhat	1	1.4	1.4	91.3
	Substantially	6	8.6	8.7	100.0
	Total	69	98.6	100.0	

Missing	System	1	1.4		
Total		70	100.0		

Make it DIFFICULT running service: Relationship with Local generic Community Nursing services

		Frequency	Percent	Valid Percent	Cumulative Percent
Valid	Not at all	39	55.7	55.7	55.7
	Somewhat	31	44.3	44.3	100.0
	Total	70	100.0	100.0	

Make it DIFFICULT running service: Relationship with Local GPs

		Frequency	Percent	Valid Percent	Cumulative Percent
Valid	Not at all	34	48.6	49.3	49.3
	Somewhat	34	48.6	49.3	98.6
	Substantially	1	1.4	1.4	100.0
	Total	69	98.6	100.0	
Missing	System	1	1.4		
Total		70	100.0		

Make it DIFFICULT running service: Other local service (specify)

		Frequency	Percent	Valid Percent	Cumulative Percent
Valid		25	35.7	35.7	35.7
	acute clinical nurse specialists	1	1.4	1.4	37.1
	care home team, education and support, neurology team, macmillan support	1	1.4	1.4	38.6
	Carers - difficult to access	1	1.4	1.4	40.0
	Clinical nurse specialist at hospital	1	1.4	1.4	41.4

	clinical nurse specialists	1	1.4	1.4	42.9
	community Macmillan	1	1.4	1.4	44.3
	Community Multi-disciplinary team	1	1.4	1.4	45.7
	continuing health care fund	1	1.4	1.4	47.1
	continuing health funding	1	1.4	1.4	48.6
	ECHO team	1	1.4	1.4	50.0
	Health funding	1	1.4	1.4	51.4
	leagues of friends	1	1.4	1.4	52.9
	local care home and pharmacies	1	1.4	1.4	54.3
	local hospital services	1	1.4	1.4	55.7
	Local pharmacy out of hours	1	1.4	1.4	57.1
	Macmillan	4	5.7	5.7	62.9
	Macmillan - mdt meetings	1	1.4	1.4	64.3
	Macmillan benefits advice	1	1.4	1.4	65.7
	Macmillan CNS	1	1.4	1.4	67.1
	Macmillan services	1	1.4	1.4	68.6
	Marie Curie	5	7.1	7.1	75.7
	Muslim burial service, chaplaincy, ICRS, Marie Curie	1	1.4	1.4	77.1
	Neuroteam	1	1.4	1.4	78.6
	Numerous other	1	1.4	1.4	80.0
	out of hours gp	1	1.4	1.4	81.4
	Professions allied to medicines	1	1.4	1.4	82.9
	Social services	11	15.7	15.7	98.6
	Social services care packages	1	1.4	1.4	100.0
	Total	70	100.0	100.0	

Make it DIFFICULT running service: Relationship with Other local service

	Frequency	Percent	Valid Percent	Cumulative Percent

Valid	Not at all	24	34.3	51.1	51.1
	Somewhat	21	30.0	44.7	95.7
	Substantially	2	2.9	4.3	100.0
	Total	47	67.1	100.0	
Missing	System	23	32.9		
Total		70	100.0		

Make it DIFFICULT running service: Too many referrals

		Frequency	Percent	Valid Percent	Cumulative Percent
Valid	Not at all	46	65.7	65.7	65.7
	Somewhat	20	28.6	28.6	94.3
	Substantially	4	5.7	5.7	100.0
	Total	70	100.0	100.0	

Make it DIFFICULT running service: Unsuitable/inappropriate referrals

		Frequency	Percent	Valid Percent	Cumulative Percent
Valid	Not at all	45	64.3	65.2	65.2
	Somewhat	23	32.9	33.3	98.6
	Substantially	1	1.4	1.4	100.0
	Total	69	98.6	100.0	
Missing	System	1	1.4		
Total		70	100.0		

Make it DIFFICULT running service: Inadequate funding

		Frequency	Percent	Valid Percent	Cumulative Percent
Valid	Not at all	24	34.3	35.3	35.3
	Somewhat	35	50.0	51.5	86.8

		Frequency	Percent	Valid Percent	Cumulative Percent
	Substantially	9	12.9	13.2	100.0
	Total	68	97.1	100.0	
Missing	System	2	2.9		
Total		70	100.0		

Make it DIFFICULT running service: Inability to recruit and retain suitable staff

		Frequency	Percent	Valid Percent	Cumulative Percent
Valid	Not at all	38	54.3	55.9	55.9
	Somewhat	28	40.0	41.2	97.1
	Substantially	2	2.9	2.9	100.0
	Total	68	97.1	100.0	
Missing	System	2	2.9		
Total		70	100.0		

Make it DIFFICULT running service: Getting Other services (e.g. social services)

		Frequency	Percent	Valid Percent	Cumulative Percent
Valid	Not at all	10	14.3	14.5	14.5
	Somewhat	48	68.6	69.6	84.1
	Substantially	11	15.7	15.9	100.0
	Total	69	98.6	100.0	
Missing	System	1	1.4		
Total		70	100.0		

Make it DIFFICULT running service: Providing services Out Of Hours

		Frequency	Percent	Valid Percent	Cumulative Percent
Valid	Not at all	32	45.7	45.7	45.7
	Somewhat	35	50.0	50.0	95.7

	Substantially	3	4.3	4.3	100.0
	Total	70	100.0	100.0	

Make it DIFFICULT running service: Inability to access necessary clinical equipment in a timely fashion

		Frequency	Percent	Valid Percent	Cumulative Percent
Valid	Not at all	31	44.3	44.3	44.3
	Somewhat	36	51.4	51.4	95.7
	Substantially	3	4.3	4.3	100.0
	Total	70	100.0	100.0	

Make it DIFFICULT running service: Inability to access anticipatory medications by injection in a timely fashion

		Frequency	Percent	Valid Percent	Cumulative Percent
Valid	Not at all	31	44.3	44.3	44.3
	Somewhat	36	51.4	51.4	95.7
	Substantially	3	4.3	4.3	100.0
	Total	70	100.0	100.0	

Make it DIFFICULT running service: Delays in administration of anticipatory medications by injection when needed

		Frequency	Percent	Valid Percent	Cumulative Percent
Valid	Not at all	27	38.6	38.6	38.6
	Somewhat	42	60.0	60.0	98.6
	Substantially	1	1.4	1.4	100.0
	Total	70	100.0	100.0	

Make it DIFFICULT running service: Geography of area (e.g. distances,parking,traffic,safety)

		Frequency	Percent	Valid Percent	Cumulative Percent
Valid	Not at all	9	12.9	12.9	12.9
	Somewhat	57	81.4	81.4	94.3
	Substantially	4	5.7	5.7	100.0
	Total	70	100.0	100.0	

Main source of income

		Frequency	Percent	Valid Percent	Cumulative Percent
Valid	NHS	17	24.3	25.8	25.8
	Charitable	44	62.9	66.7	92.4
	Donations	3	4.3	4.5	97.0
	Other	2	2.9	3.0	100.0
	Total	66	94.3	100.0	
Missing	System	4	5.7		
Total		70	100.0		

Other main source of income (specify)

		Frequency	Percent	Valid Percent	Cumulative Percent
Valid		58	82.9	82.9	82.9
	17% NHS	1	1.4	1.4	84.3
	2/3 invome from NHS (not overheads); 1/3 charity	1	1.4	1.4	85.7
	30% NHS funding	1	1.4	1.4	87.1
	33% NHS	1	1.4	1.4	88.6
	48% NHS funded	1	1.4	1.4	90.0
	50% NHS/50%charity	1	1.4	1.4	91.4
	65% NHS	1	1.4	1.4	92.9

	80% charity	1	1.4	1.4	94.3
	Continuing health care funding	1	1.4	1.4	95.7
	Fully commissioned	1	1.4	1.4	97.1
	NHS & Local Authority	1	1.4	1.4	98.6
	NHS until 2015, also charitable and donations	1	1.4	1.4	100.0
	Total	70	100.0	100.0	

Other source of income: NHS

		Frequency	Percent	Valid Percent	Cumulative Percent
Valid	Ticked	60	85.7	100.0	100.0
Missing	System	10	14.3		
Total		70	100.0		

Other source of income: Charitable

		Frequency	Percent	Valid Percent	Cumulative Percent
Valid	Ticked	67	95.7	100.0	100.0
Missing	System	3	4.3		
Total		70	100.0		

Other source of income: Donations

		Frequency	Percent	Valid Percent	Cumulative Percent
Valid	Ticked	48	68.6	100.0	100.0
Missing	System	22	31.4		
Total		70	100.0		

Other source of income: Other

		Frequency	Percent	Valid Percent	Cumulative Percent
Valid	Ticked	48	68.6	100.0	100.0
Missing	System	22	31.4		
Total		70	100.0		

Other source of income (specify)

		Frequency	Percent	Valid Percent	Cumulative Percent
Valid		22	31.4	31.4	31.4
	10% grants; 42.5% hospice shops; occasional legacy	1	1.4	1.4	32.9
	14 hospice shops, legacies, in memoriam giving	1	1.4	1.4	34.3
	Charity shops	1	1.4	1.4	35.7
	Fundraising, hospice shops	1	1.4	1.4	37.1
	H@H hospice shop; educational packages	1	1.4	1.4	38.6
	Hospice funding	1	1.4	1.4	40.0
	Hospice shops	19	27.1	27.1	67.1
	Hospice shops x5; usual fundraising	1	1.4	1.4	68.6
	Hospice shops * 8	1	1.4	1.4	70.0
	Hospice shops and education/training programme	1	1.4	1.4	71.4
	Hospice shops and grants eg Macmillan	1	1.4	1.4	72.9
	Hospice shops and tea shop	1	1.4	1.4	74.3
	Hospice shops x 30	1	1.4	1.4	75.7
	Hospice shops x 7	1	1.4	1.4	77.1
	Hospice shops, covenants	1	1.4	1.4	78.6

	Hospice shops, education	1	1.4	1.4	80.0
	Hospice shops, education has small income	1	1.4	1.4	81.4
	Hospice shops, education-paid courses, projects	1	1.4	1.4	82.9
	Hospice shops, fundraising	1	1.4	1.4	84.3
	Hospice shops, health lottery	1	1.4	1.4	85.7
	Hospice shops, league of friends	1	1.4	1.4	87.1
	Hospice shops, legacies	1	1.4	1.4	88.6
	Hospice shops/cafes	1	1.4	1.4	90.0
	Large fund raising teams	1	1.4	1.4	91.4
	Legacies	1	1.4	1.4	92.9
	Legacies, grants, hospice shops	1	1.4	1.4	94.3
	Legacy	1	1.4	1.4	95.7
	Legacy and shops	1	1.4	1.4	97.1
	Local authority	1	1.4	1.4	98.6
	shops, fundraising, events	1	1.4	1.4	100.0
	Total	70	100.0	100.0	

Extent of NHS funding

		Frequency	Percent	Valid Percent	Cumulative Percent
Valid	None	7	10.0	10.3	10.3
	Secondary source	44	62.9	64.7	75.0
	Main source	17	24.3	25.0	100.0
	Total	68	97.1	100.0	
Missing	System	2	2.9		
Total		70	100.0		

```
*** Evidence 24/7 service by DN 24h service.
cro tab=evidence247 by dnservices.
```
